# SbWRKY75- and SbWRKY41-mediated jasmonic acid signaling regulates baicalin biosynthesis

**DOI:** 10.3389/fpls.2023.1213662

**Published:** 2023-06-21

**Authors:** Shiyuan Fang, Chen Zhang, Shi Qiu, Ying Xiao, Kaixian Chen, Zongyou Lv, Wansheng Chen

**Affiliations:** ^1^ The State Administration of Traditional Chinese Medicine (SATCM) Key Laboratory for New Resources & Quality Evaluation of Chinese Medicine, Institute of Chinese Materia Medica, Shanghai University of Traditional Chinese Medicine, Shanghai, China; ^2^ Institute of Chinese Materia Madica, Shanghai University of Traditional Chinese Medicine, Shanghai, China; ^3^ Department of Pharmacy, Changzheng Hospital, Second Military Medical University, Shanghai, China

**Keywords:** JA, Baicalin, *Scutellaria baicalensis* Georgi, SbWRKY75, SbWRKY41

## Abstract

**Introduction:**

*Scutellaria baicalensis* Georgi is a traditional Chinese medicinal plant with broad pharmacological activities whose main active ingredient is the flavonoid baicalin. Given its medicinal value and increasing market demand, it is essential to improve the plant’s baicalin content. Flavonoid biosynthesis is regulated by several phytohormones, primarily jasmonic acid (JA).

**Methods:**

In this study, we conducted transcriptome deep sequencing analysis of *S. baicalensis* roots treated with methyl jasmonate for different durations (1, 3, or 7 hours). Leveraging weighted gene co-expression network analysis and transcriptome data, we identified candidate transcription factor genes involved in the regulation of baicalin biosynthesis. To validate the regulatory interactions, we performed functional assays such as yeast one-hybrid, electrophoretic mobility shift, and dual-luciferase assays.

**Results:**

Our findings demonstrated that SbWRKY75 directly regulates the expression of the flavonoid biosynthetic gene *SbCLL-7*, whereas SbWRKY41 directly regulates the expression of two other flavonoid biosynthetic genes, *SbF6H* and *SbUGT*, thus regulating baicalin biosynthesis. We also obtained transgenic *S.baicalensis* plants by somatic embryo induction and determined that overexpressing SbWRKY75 increased baicalin content by 14%, while RNAi reduced it by 22%. Notably, SbWRKY41 indirectly regulated baicalin biosynthesis by modulating the expression of *SbMYC2.1, SbJAZ3* and *SbWRKY75*.

**Discussion:**

This study provides valuable insights into the molecular mechanisms underlying JA-mediated baicalin biosynthesis in *S. baicalensis*. Our results highlight the specific roles of transcription factors, namely SbWRKY75 and SbWRKY41, in the regulation of key biosynthetic genes. Understanding these regulatory mechanisms holds significant potential for developing targeted strategies to enhance baicalin content in *S. baicalensis* through genetic interventions.

## Introduction

Chinese skullcap (*Scutellaria baicalensis* Georgi), a plant used in traditional Chinese medicine, is renowned for its diverse pharmacological activities, such as heat-clearing, detoxification, diuresis, sore-throat relief, and anti-inflammatory effects, which are primarily attributed to its flavonoid constituents, including baicalein, baicalin, wogonin, and wogonoside (Zhao et al., 2016a). Zhao et al. discovered a specialized flavone biosynthetic pathway in *S. baicalensis* (Zhao et al., 2016b). This pathway encompasses the enzymatic conversion of cinnamoyl-CoA to cinnamoyl, subsequent catalytic transformation of pinocembrin to chrysin mediated by flavone synthase II (FNSII), and further conversions of chrysin into wogonin and baicalein (Zhao et al., 2019). The elucidation of this pathway establishes a crucial foundation for comprehending the biosynthesis of these essential flavonoid compounds in *S. baicalensis*. Given the medicinal value and increasing market demand of *S. baicalensis*, enhancing the content of active ingredients in this plant is of great economical and pharmacological importance. Previous studies have shown that the exogenous application of phytohormones, especially jasmonic acid (JA), stimulates the accumulation of baicalin in *S. baicalensis* (Xu et al., 2010). However, the mechanism by which JA increases baicalin biosynthesis is likely to involve multiple pathways and factors that are not well understood.

Baicalin biosynthesis and accumulation in *S. baicalensis* are known to be tightly regulated by various internal and external signals, although the underlying mechanisms remain largely unclear (Wang et al., 2022b). Among these signals, JA and its derivatives are plant stress hormones that play key roles in regulating flavonoid biosynthesis, root growth, and defense against pathogen infection and insect attack (Zhu et al., 2022). Studies on Arabidopsis (*Arabidopsis thaliana*) have revealed that JA regulates anthocyanin biosynthesis through the activation of transcription factors (TFs) (Shan et al., 2009). The TF MYC2 and JASMONATE-ZIM-DOMAIN PROTEIN (JAZ) repressors have emerged as the master regulators of most aspects of the JA signaling pathway in Arabidopsis (Kazan and Manners, 2013). Indeed, MYC2 is an important transcription factor that activates the expression of flavonoid biosynthetic genes by binding to specific E-box elements in their promoter regions or by forming complexes with other TFs (Van Moerkercke et al., 2019). In the absence of JA, JAZ repressors directly bind to MYC2 to form a repressive transcriptional complex. In the presence of JA, CORONATINE INSENSITIVE1 (COI1) recruits JAZ repressors, leading to their ubiquitination and subsequent degradation through the 26S proteasome, which releases MYC2 that induces JA responses (Chini et al., 2007; Thines et al., 2007).

The sophisticated transcriptional regulation of JA response in plants is mediated by various TF families, including APETALA2 (AP2)/ETHYLENE-RESPONSE FACTOR (ERF), basic helix-loop-helix (bHLH), MYB, and WRKY (Zhao et al., 2020; Huang et al., 2022; Liu et al., 2023; Yuan et al., 2023). Among these TF families, the WRKY family has emerged as a crucial player in the regulation of biosynthetic pathways for various bioactive compounds. Notably, studies conducted in *S. baicalensis* have provided evidence for a close association between WRKY TFs and the accumulation of anthocyanins (Zhang et al., 2022). Furthermore, the expression of *NnWRKY40a* and *NnWRKY40b*, two WRKY transcription factor genes cloned from lotus (*Nelumbo nucifera*), is significantly induced by JA and promotes BIA biosynthesis by modulating the expression of BIA biosynthetic genes (Li et al., 2022). Similarly, TcWRKY26 in Chinese yew (*Taxus chinensis*) directly binds to the W-box in the *DBAT* (10-deacetylbaccatin III-10β-O-acetyltransferase gene) promoter to activate its expression, with the TcWRKY26–TcJAV3 complex defining another JA signal transduction pathway that effectively regulates taxol biosynthesis (Chen et al., 2022). The diverse roles of WRKY family members in regulating the biosynthesis of different bioactive compounds indicate their significance in the JA response of plants.

In this study, we investigated the transcriptional regulation of baicalin biosynthesis in the roots of *S. baicalensis* following treatment with methyl jasmonate (MeJA). To this end, we collected root samples at four different time points and performed a transcriptome deep sequencing (RNA-seq) analysis, followed by weighted gene co-correlation network analysis (WGCNA) to construct a TF-gene regulatory network. By analyzing the regulatory network, we identified key TF genes likely to be involved in the JA-mediated regulation of baicalin biosynthesis in *S. baicalensis*. Our results shed light on the complex signaling and transcriptional cascade underlying the biosynthesis of this important flavone compound.

## Materials and methods

### Plant materials and MeJA treatment

All plant materials were cultivated in a greenhouse under a controlled environment of 22°C with a 16-h light/8-h dark photoperiod. For JA response analysis, 1-month-old *S. baicalensis* seedlings were transferred to an aqueous solution containing 200 μM MeJA. The roots of the seedlings were collected at four time points, including 0, 1, 3, and 7 h of MeJA treatment.

### RNA-seq and data analysis

Roots of *S. baicalensis* seedlings subjected to MeJA treatment for 0, 1, 3, and 7 h were used for transcriptome analysis. Total RNA was extracted using TRIzol reagent (Invitrogen, Carlsbad, CA, USA) according to the manufacturer’s instructions. LC-Bio Technology Co., Ltd. (Hangzhou, China) generated the sequencing libraries and conducted sequencing. Reference transcriptome data are deposited in the Sequence Read Archive (SRA) under accession number SRA: PRJNA961700.

### Weighted gene co-expression network analysis

WGCNA was conducted using the OmicStudio tools available at https://www.omicstudio.cn/tool/WGCNA. A pairwise Pearson’s correlation matrix was constructed and transformed into a weighted matrix. To create a topological overlap matrix, a soft threshold of 0.85 was applied. Module-trait associations were estimated to identify the relationship between modules and various treatment stages, by calculating the correlation between the module eigengene and the flavone contents at each infection stage. This approach facilitated the identification of expression modules that were highly correlated with the flavone contents. The networks were visualized using Cytoscape software.

### Identification of *S. baicalensis* TFs, MYC2 and JAZ genes

*S. baicalensis* transcription factor genes were identified through a comparative analysis of transcripts assembled from RNA-seq data against the PlantTFDB database using the BlastX algorithm. Candidate *SbMYC2* genes were further selected by BLAST using the *AtMYC2* sequence as query. Additionally, the hidden Markov model (HMM) profiles PF06200 (TIFY domain) and PF09425 (Jas motif) of the JAZ family were retrieved from the Pfam database, and the transcriptome sequence was subjected to hmmsearch analysis to identify candidate *JAZ* genes. Finally, a phylogenetic tree was constructed with all candidate TFs using MEGA7 software to facilitate visualization and interpretation of the results.

### Plant transformation *via* somatic embryogenesis

The coding region of *SbWRKY75* was cloned into the pHB vector to generate the pHB : *SbWRKY75* overexpression vector. A fragment of the *SbWRKY75* coding sequence (bp 9–293) was selected for RNA interference (RNAi) and cloned into the intermediate vector pENTR. The target fragment was then recombined into the pHELLSGATE vector *via* LR Clonase to obtain the RNAi interference construct pHELLSGATE : *SbWRKY75* (**Figure S2**). The pHB : *SbWRKY75* and pHELLSGATE : *SbWRKY75* clones were subsequently transformed into Agrobacterium (*Agrobacterium tumefaciens*) strain GV3101.

The seeds were washed with running water for a duration of 30 minutes, followed by a cold storage period of 72 hours at 4°C. Subsequently, the seeds were immersed in 70% ethanol for 30 seconds and rinsed thrice with sterile water to ensure surface sterilization. A 1% sodium hypochlorite treatment was then administered for 15 minutes, followed by three additional rinses with sterile water. The surface-sterilized seeds were then inoculated onto Murashige and Skoog (MS) medium to facilitate the cultivation of sterile seedlings. Leaf and stem segments were excised from *S. baicalensis* plantlets and used as explants to induce somatic embryogenesis. The explants were immersed in Agrobacterium suspension (OD=0.6) with uniform shaking in the dark for 10 min. After being blotted dry onto sterile tissue paper, the explants were transferred to MS medium with 10.0 mg/L 1-naphthaleneacetic acid. The inoculated explants were selected on selective medium containing 200 mg/L timentin and cultivated at 25°C in the dark to induce somatic embryogenesis. After the formation of somatic embryos, they were subjected to an alternating light/dark cycle of 16 hours of light and 8 hours of darkness under a light intensity of 2,000 lux. The seedlings were then transferred to 1/2 MS medium to induce root formation. The rooted seedlings were transferred to flower discs containing substrate for further cultivation. Genomic DNA was extracted from the young leaves of transgenic *S. baicalensis* seedlings for confirmation of transformation.

### Yeast one-hybrid assays

The promoter fragments of baicalin biosynthesis pathway genes were cloned into the pLacZ vector as bait. The coding sequences of *SbWRKY75*, *SbWRKY41*, and *SbRAP2.1* were recombined into the pB42AD vector as prey. The primer sequences are listed in **Table S1**. Different combinations of vectors were co-transformed into yeast strain EGY48. Transformants were selected on synthetic defined (SD) medium lacking tryptophan and leucine. Positive yeast colonies were grown for 3 days at 30°C, collected, and resuspended in sterile water, before being spotted onto an X-gal medium plate, which was then incubated at 30°C for 24 h. Negative controls were established using the empty pLacZ and pB42AD plasmids.

### Recombinant protein production and purification

The coding sequences of *SbWRKY75*, *SbWRKY41*, and *SbRAP2.1* were cloned into the pET32a expression vector to produce His-tagged fusion proteins. The sequences of the primers used for cloning are provided in **Table S1**. The resulting plasmids were transformed into *E. coli* BL21 cells, and the production of recombinant proteins was induced by adding 0.1 mM IPTG at 18°C for 12 h. The bacterial cells pelleted by centrifugation and lysed using a low-temperature ultra-high-pressure continuous flow cell disrupter (JNBIO). The supernatant was purified using Ni-NTA resin (Qiagen, Cat. No. 30210), and the bound proteins were eluted with 200 mM imidazole and subsequently concentrated using a Millipore (Merck, Cat. No. UFC903024) ultrafiltration device.

### EMSAs

To investigate whether SbWRKY75, SbWRKY41, and SbRAP2.1 can bind to the promoters of their target genes, electrophoretic mobility shift assays (EMSAs) were conducted. The probes used in EMSA were labeled with digoxigenin; their sequences are listed in **Table S1**. The EMSA reactions were performed using a DIG Gel Shift Kit, 2nd Generation, following the manufacturer’s instructions (Roche). The reaction products were loaded onto a 5% polyacrylamide gel and electrophoresed at 80 V for 90 min. The DNA-protein complexes were transferred to a nylon membrane for 60 min at 300 mA and crosslinked at 120 MJ. Probe detection was carried out according to the protocol provided by Roche.

### Subcellular localization

The coding sequences of *SbWRKY75* and *SbWRKY41* without a stop codon were individually cloned into the fusion YFP vector pHB through one-step cloning. The resulting constructs were transformed into Agrobacterium strain GV3101 and infiltrated into *Nicotiana benthamiana* leaves using needleless syringes. YFP fluorescence in lower epidermal cells was observed 48 h to 72 h after Agrobacterium infiltration using a confocal microscope (Leica TCS SP8). Primers used are listed in **Table S1**.

### Dual-luciferase assay

To perform the dual-luciferase reporter gene assay (dual-LUC), the promoter sequences of *SbCLL-7*, *SbF6H*, and *SbUGT* were cloned individually into pGreen II 0800 by one-step cloning (Lv et al., 2019). The resulting constructs were transformed into Agrobacterium strain GV3101. *N. benthamiana* leaves were infiltrated with the Agrobacterium cultures using needleless syringes. The leaves were collected 48 h after infiltration for dual-LUC assays, which were performed using a Dual-Luciferase Reporter Assay System according to the manufacturer’s instructions (Promega). Primers used are listed in **Table S1**.

### Measurements of flavone content

The quantification of baicalin, baicalein, wogonoside, and wogonin was carried out using an Agilent 6410 Triple Quad LC/MS system. Root samples of *S. baicalensis* were collected and homogenized to obtain a fine powder, which was then subjected to ultrasonic extraction with 70% (v/v) ethanol at 60°C for 1 h. The obtained extracts were centrifuged at 10,000 g at 4°C for 15 min and subsequently filtered through a Millipore Millex-HV filter (Merck Millipore, Billerica, MA, USA). To determine the flavone content, an absolute quantification method with external standards was employed. Calibration curves were constructed using standard solutions of baicalin, baicalein, wogonoside, and wogonin at known concentrations. The analytical parameters of the Agilent 6410 Triple Quad LC/MS were as follows: column, Waters T3 column (2.1 mm × 100 mm, 3 μm); oven temperature, 350°C. The mobile phase contained D: water (containing 0.1% [v/v] formic acid); C: acetonitrile; flow rate, 0.3 mL/min; column temperature, 50°C; and injection volume, 5 μL.

### Reverse-transcription quantitative PCR

Total RNA was extracted from the roots of control *S. baicalensis* seedlings and those of seedlings treated with 200 µM MeJA for 0, 1, 3, or 7 h using TRIzol Reagent. Total RNA samples was then reverse-transcribed to first-strand cDNA using PrimeScript II RTase (Takara cat. no. 6110A). Primer pairs were designed using Primer Premier 5 software. Quantitative PCR was performed on a QuantStudio 3 System following the manufacturer’s protocol. The relative expression levels of target genes were calculated using the 2^–ΔΔCt^ method with *SbACT7* as the reference gene. The primer sequences are available in **Table S1**.

### Statistical analysis

All boxplots, bar graphs, and connecting lines were generated using Origin 2018 Software. The error bars in the figures represent the standard deviation of the means obtained from three independent biological replicates.

## Results

### MeJA treatment influences flavone biosynthesis

**Figure 1:**
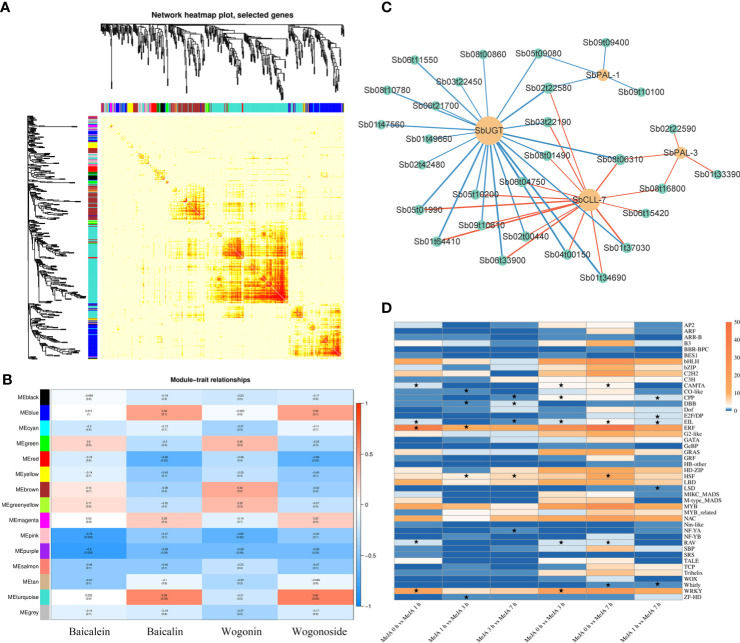
Co-expression network analysis and transcription factor regulation of baicalin biosynthesis in *S. baicalensis* with MeJA treatment. **(A)** WGCNA-based clustering analysis of samples and construction of the co-expression modules. Each module is indicated by different colors in the row; **(B)** Heatmap representation of the correlation between WGCNA modules and flavone contents. Darker red represents a high positive correlation; darker blue indicates a high negative correlation; **(C)** Analysis and visualization of the correlation between the top 30% transcription factor genes from the ‘Mebrown’ module and baicalin biosynthesis genes; **(D)** Heatmap representation of the expression levels of transcription factor gene families. The color shows the number of differentially expressed genes (DEGs) in a particular gene family. The asterisks indicate the top five transcription factor gene families with high numbers of DEGs between groups.

To investigate the effects of MeJA treatment on baicalin biosynthesis in *S. baicalensis* roots, we treated plants with an aqueous MeJA solution for 0, 1, 3, or 7 h (**Figure 1A**). We determined that the levels of baicalein, baicalin, wogonin, and wogonoside change rapidly over the course of the treatment (**Figure 1B**). Specifically, the levels of baicalein, baicalin, wogonin, and wogonoside significantly decreased by 54%, 96%, 31% and 97%, respectively, after 1 h of MeJA treatment compared to the 0-h samples. However, after 7 h of MeJA exposure, the levels of these compounds had increased to reach 95% (baicalein), 19% (baicalin), 113% (wogonin), and 13% (wogonoside) of their levels in the 0-h samples. These results suggest that the treatment resulted in a substantial decrease in flavone glycoside content, followed by a slow recovery. Conversely, the decrease in glycoside content was modest and this essentially returned to pre-treatment levels after 7 h of MeJA treatment. Therefore, MeJA treatment has a significant influence on the flavone contents of *S. baicalensis* roots.

We examined the expression of various genes known to encode enzymes involved in baicalin biosynthesis in *S. baicalensis* (**Figure 1C**). These genes consisted of *SbPAL-1* (*Phenylalanine ammonia-lyase 1*), *SbPAL-2*, *SbPAL-3* (Park et al., 2012), *SbCLL-7* (*Cinnamate-CoA ligase 7*), *SbCHS-2* (*Chalcone synthase 2*), *SbCHI* (*Chalcone isomerase*), *SbFNSII-2* (*Flavone synthase II-2*), *SbF6H* (*Flavonoid 6-hydroxylase*) (Zhao et al., 2016b), *SbUGT* (*UDP-Glucosyl transferase*) (Nagashima et al., 2000) and *SbGUS* (*BETA-D-Glucuronidase*) (Ikegami et al., 1995). We observed significant changes in the expression levels of these genes in seedling roots upon treatment with exogenous MeJA, in agreement with the observed alterations in flavone contents described above. These findings suggest that the MeJA-induced regulation of flavone biosynthesis in *S. baicalensis* is mediated through transcriptional modulation of various key biosynthetic genes in this pathway.

### Weighted correlation network analysis

**Figure 2:**
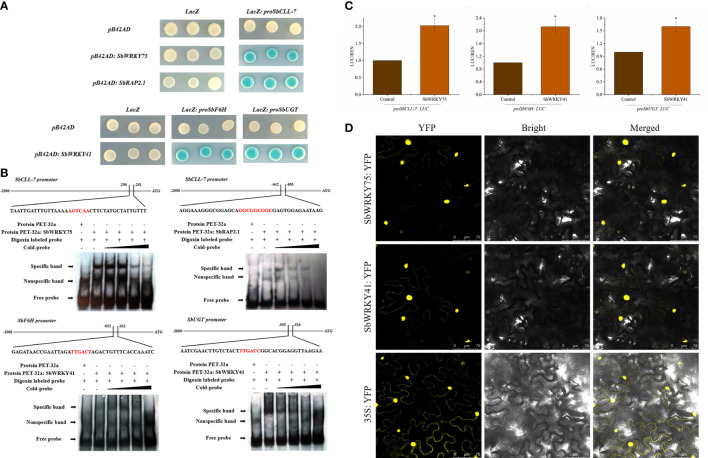
Detection of binding between the SbWRKY75, SbWRKY41 and SbRAP2.1 and their target promoters by Y1H assay **(A)** and EMSA **(B)**; **(C)** Dual-LUC assay showing that SbWRKY75 and SbWRKY41 activate the transcription of *SbCLL-7* and *SbF6H*, *SbUGT* in *N. benthamiana*. The *YFP* effector construct was used as a negative control, and the LUC/REN ratios for *YFP* were set to 1; **(D)** Subcellular localization of SbWRKY75 and SbWRKY41 in *N. benthamiana* leaves.

We employed the WGCNA R package to identify modules of co-expressed genes in response to MeJA treatment. With a soft minimal threshold for Pearson’s correlation coefficient of 0.85, we identified 15 co-expression modules, as defined on differential expression of their constituent genes, with each module being indicated by a different color (**Figure 2A**, **Table S2**). We clustered the consensus module eigengenes and visualized them to analyze module-phenotype correlations. Among the different gene expression modules, we found that the ‘MEturquoise’ module exhibited a strong and positive correlation with both baicalin and wogonoside contents, suggesting that the genes within this module play important roles in regulating the biosynthesis of these two compounds. Additionally, the ‘MEbrown’ module showed a strong and positive correlation specifically with wogonin content, indicating that the genes within this module may be involved in the specific biosynthesis of wogonin (**Figure 2B**). KEGG pathway enrichment analysis revealed that the ‘MEturquoise’ module does not contain genes directly involved in the phenylpropanoid pathway. Therefore, we focused on the ‘MEbrown’ module for subsequent analysis (**Figure S1**). We selected genes encoding baicalin biosynthetic enzymes and TFs within the top 30% genes from the ‘MEbrown’ module as candidate genes and identified key transcription factor genes regulating baicalin biosynthesis through correlation analysis (**Figure 2C**).

To investigate the TFs involved in regulating baicalin biosynthesis during MeJA processing, we looked at the number of differentially expressed TF genes in different TF gene families at different time points. We observed that the bHLH, ERF, MYB, NAC, and WRKY families are represented by the highest numbers of differentially expressed genes, with the proportion of differentially expressed TF genes in the ERF and WRKY families being particularly high (**Figure 2D**). Notably, TFs in the WRKY and ERF families have been shown to play important roles in flavonoid biosynthesis (Lloyd et al., 2017; Wu et al., 2022). Therefore, we chose five WRKY TF genes and four ERF TF genes with a *p*<0.01, as determined by Pearson’s correlation analysis with baicalin biosynthesis pathway genes, from the top 30% central TF genes in the ‘MEbrown’ module defined by WGCNA analysis. We investigated the function of these selected TF genes below.

### SbWRKY75, SbWRKY41, and SbRAP2.1 regulate baicalin biosynthesis

**Figure 3:**
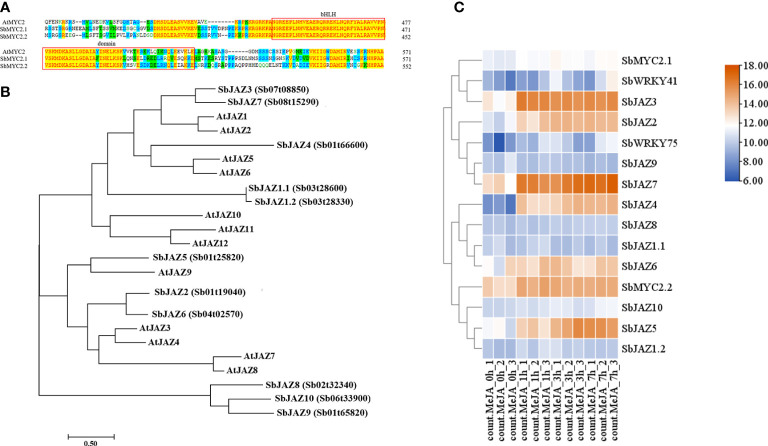
**(A)** Sequence alignment of SbMYC2 and AtMYC2; **(B)** Phylogenetic analysis of SbJAZ and AtJAZ proteins; **(C)** Heatmap representation showing the expression levels of *SbMYC2*s, *SbJAZ*s, *SbWRKY75* and *SbWRKY41* in control *S. baicalensis* roots and after MeJA treatment. Each value was transformed using a Log2 conversion.

We cloned the promoter sequence of several baicalin biosynthesis pathway genes into the pLacZ vector. To identify TFs potentially binding to these promoter sequences, we conducted a yeast one-hybrid (Y1H) assay using the above five WRKY TF genes and four ERF TF genes. As indicated by the blue color of the corresponding yeast colonies harboring the relevant constructs, we determined that SbWRKY75 (Sb01t34690) bound to the *SbCLL-7* promoter, while SbWRKY41 (Sb06t15420) bound to the *SbF6H* and *SbUGT* promoters, and SbRAP2.1 (Sb08t10780) bound to the *SbCLL-7* promoter (**Figure 3A**).

We validated the above results by conducting an electrophoretic mobility shift assay (EMSA) and a dual-luciferase (LUC) assay. To this end, we produced recombinant SbWRKY75 and SbRAP2.1 fused to a thioredoxin tag and incubated the purified proteins with digoxigenin-labeled probes for the *SbCLL-7* promoter. We detected specific binding of both recombinant proteins to labeled probes, as evidenced by the lower mobility of the probes from the corresponding target genes (**Figure 3B**). Importantly, this lower mobility was gradually competed by incubation with unlabeled probe (**Figure 3B**). In the dual-LUC assay, using the reporter constructs *proSbCLL-7:LUC*, *proSbF6H:LUC*, and *proSbUGT : LUC*, we observed that co-expression of SbWRKY75 with the *proSbCLL-7:LUC* reporter construct resulted in a 103% increase in the LUC/REN ratio (firefly to Renilla luciferase activity). Additionally, co-expression of SbWRKY41 with the *proSbF6H:LUC* and *proSbUGT : LUC* reporter constructs resulted in a 113% increase and a 60% increase in the LUC/REN ratio, respectively (**Figure 3C**).

We also generated constructs consisting of the *SbWRKY75* or *SbWRKY41* coding sequence cloned in-frame with the sequence of yellow fluorescent protein (*YFP*) and driven by the cauliflower mosaic virus (CaMV) *35S* promoter to establish the subcellular localization of these two TFs. We detected YFP fluorescence for *SbWRKY75:*YFP and *SbWRKY41:*YFP mainly in the nucleus of *N. benthamiana* leaf cells, indicating that SbWRKY75 and SbWRKY41 localize to the nucleus (**Figure 3D**).

### SbWRKY75 accelerates baicalin biosynthesis in *S. baicalensis*

**Figure 4:**
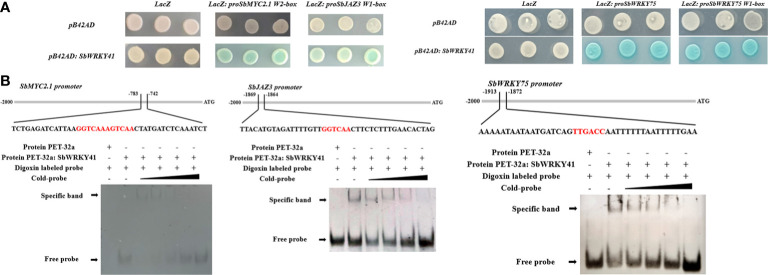
Y1H **(A)** and EMSA **(B)** results showing the binding of SbWRKY41 to the W2-box of the *SbMYC2.1* promoter, the W1-box of the *SbJAZ3* promoter, and the W1-box of the *SbWRKY75* promoter.

To investigate the effect of SbWRKY75 on the baicalin biosynthesis pathway in *S. baicalensis*, we transiently overexpressed *SbWRKY75 via* Agrobacterium-mediated transient transformation. We detected a significant 73% increase in *SbWRKY75* transcript levels in the roots of these transiently overexpressing plants (**Figure 4A**). Conversely, we measured about a 43% decrease in *SbWRKY75* transcript levels in *S. baicalensis* plants transiently expressing an RNAi construct specific for *SbWRKY75* (**Figure 4B**). We then analyzed the relative expression levels of ten baicalin biosynthesis genes in these *SbWRKY75* overexpression and RNAi plants. The expression levels of *SbCLL-7*, *SbPAL-1*, *SbPAL-2*, *SbCHS-2*, *SbFNSII-2*, *SbF6H*, and *SbUGT* were significantly upregulated in the *SbWRKY75* overexpression seedlings, showing approximately 0.9-, 1.1-, 1.77-, 0.45-, 7.70-, 1.64-, and 0.17-fold increases, respectively. Conversely, the expression levels of *SbCLL-7*, *SbPAL-1*, *SbCHS-2*, *SbCHI*, *SbUGT*, and *SbGUS* were approximately 84%, 34%, 21%, 59%, 48%, and 84% lower in the *SbWRKY75* RNAi seedlings compared to wild-type *S. baicalensis* seedlings.

We also measured baicalin contents in the roots of *SbWRKY75* overexpression and RNAi seedlings (**Figure 4C**). Compared to those in wild-type *S. baicalensis* seedlings, we detected baicalin and wogonoside levels that were higher by 14% and 13%, respectively, in *SbWRKY75* overexpression seedlings, whereas the levels of these compounds decreased by 22% and 19% in the *SbWRKY75*-RNAi seedlings. These results further support a role for SbWRKY75 in regulating baicalin accumulation.

### SbWRKY41 is essential for JA-induced baicalin biosynthesis

**Figure 5:**
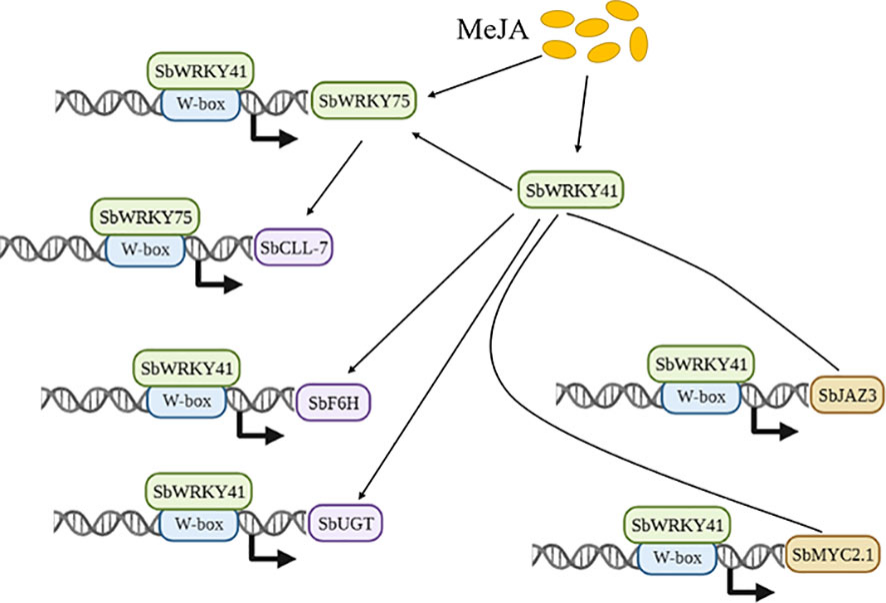
A simplified view of two JA-related SbWRKYs actively regulating flavonoid biosynthesis.

The altered expression of baicalin biosynthesis genes in MeJA-treated roots suggested that transcriptional regulation contributes to JA-induced baicalin biosynthesis. MYC TFs and JAZ proteins are critical regulators of the JA signaling pathway, making them potential targets for investigation in *S. baicalensis* (Li et al., 2017). Analysis of SbMYC2.1 and SbMYC2.2 revealed the presence of conserved domains shared with their homologs in Arabidopsis (**Figure 5A**). In addition, we identified three and one W-boxes in their promoters, respectively, raising the possibility that their transcription may be regulated *via* direct binding by WRKY TFs. Moreover, using HMMsearch, we identified 11 members of the JAZ family in *S. baicalensis* and reconstructed a phylogenetic tree together with JAZ proteins from Arabidopsis (**Figure 5B**). This analysis revealed that SbJAZ3 is similar to SbJAZ7, AtJAZ1, and AtJAZ2, while SbJAZ4 was more similar to AtJAZ5 and AtJAZ6. Additionally, SbJAZ5 clustered together with AtJAZ9, suggesting that these TFs may exert similar functions in plants. However, SbJAZ8, SbJAZ9, and SbJAZ10 were grouped together in a distinct branch, indicating that they may have undergone unique changes in their evolution and have different biological functions.

Turning to our RNA-seq analysis of gene expression in the roots of *S. baicalensis* following MeJA treatment, we observed similar upregulation of gene expression patterns after MeJA treatment for *SbWRKY75*, *SbJAZ9*, *SbJAZ7*, *SbWRKY41*, *SbMYC2.1*, and *SbJAZ3* (**Figure 5C**), indicating that SbWRKY75 and SbWRKY41 are necessary for JA-induced baicalin biosynthesis and suggesting that they may regulate the transcription of both *SbMYC2*s and *SbJAZ*s.

**Figure 6:**
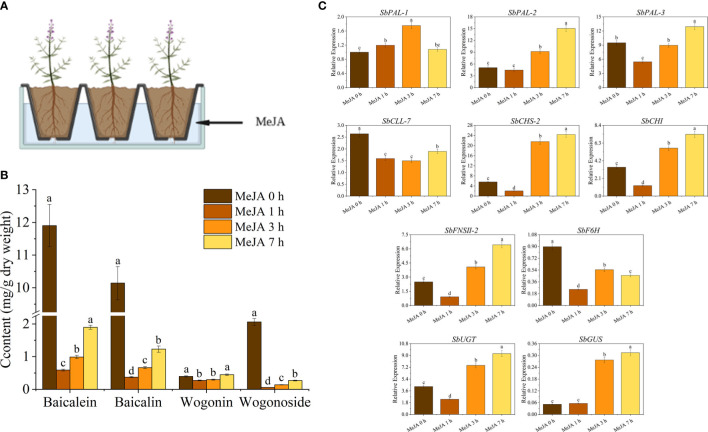
MeJA treatment influenced flavone production and baicalein biosynthesis genes in roots of *S. baicalensis* seedlings. **(A)** Schematic diagram of MeJA treatment of the roots of 1-month-old *S. baicalensis* seedlings; **(B)** Changes in flavonoid contents in roots of *S. baicalensis* seedlings treated with MeJA of 0, 1, 3, and 7 h; **(C)** RT-qPCR analysis of the expression of baicalein biosynthetic pathway-related genes in response to MeJA treatment.

We investigated the binding of SbWRKY75 to the promoters of *SbJAZ7* and *SbJAZ9*, and of SbWRKY41 to the promoters of *SbMYC2.1* and *SbJAZ3* using a Y1H assay. We tested various promoter fragments, finding that SbWRKY41 binds to the W2-box motif in the *SbMYC2.1* promoter and to the W1-box motif in the *SbJAZ3* promoter (**Figure 6A**). To confirm this interaction, we purified full-length recombinant SbWRKY41 and performed an EMSA using digoxigenin-labeled fragments containing the W2-box motif from the *SbMYC2.1* promoter and the W1-box from the *SbJAZ3* promoter motif as probes. We determined that SbWRKY41 binds to the W-box motifs of the *SbMYC2.1* and *SbJAZ3* promoters; importantly, binding decreased when unlabeled probes were added as competitors (**Figure 6B**). This finding indicates that SbWRKY41 specifically binds to the W-box motifs in the *SbMYC2.1* and *SbJAZ3* promoters.

The enrichment of W-box motifs in *WRKY* promoters suggests that WRKY TFs can regulate their own expression or cross-regulate with other WRKY TFs (Maleck et al., 2000). To investigate the potential regulatory relationship between SbWRKY75 and SbWRKY41, we conducted Y1H and EMSA experiments. We established that SbWRKY41 can modulate the expression of *SbWRKY75* and may function upstream of SbWRKY75 (**Figure 6B**).

## Discussion

**Figure 7:**
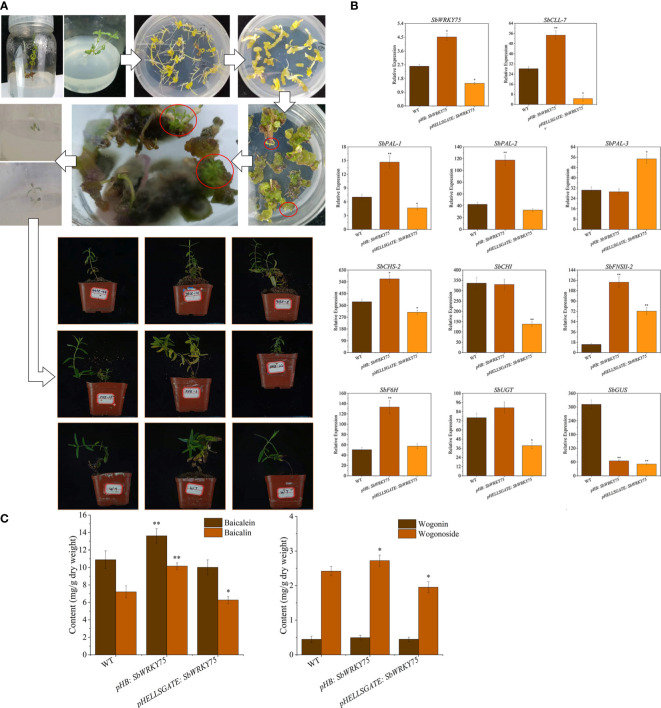
Transgenic *S. baicalensis* seedlings were obtained *via* somatic embryogenesis. **(A)** Schematic overview of somatic embryogenesis in *S. baicalensis*; **(B)** Relative expression levels of flavonoid biosynthetic genes and **(C)** flavonoid contents in transgenic *S. baicalensis* overexpressing (pHB) or silenced (pHELLSGATE) for *SbWRKY75*.

*S. baicalensis* has been a staple of traditional Chinese medicine for over two millennia. JA has been shown to play a role in enhancing flavone production in a number of medicinal plants, including *S. baicalensis* (Zhou et al., 2012; Horbowicz et al., 2021; Yao et al., 2022). For instance, treating three-week-old cultured roots of *S. baicalensis* with MeJA resulted in a doubling of total flavone content in roots, with greater increases seen for the aglycones baicalein and wogonin, which increased 230% and 330%, respectively (Kuzovkina et al., 2005). However, the molecular mechanism underlying JA-induced baicalin production and expression of the relevant biosynthesis pathway genes has remained elusive. In this study, we discovered that JA-activated SbWRKY75 regulates *SbCLL-7* transcription, while SbWRKY41 regulates *SbF6H* and *SbUGT* transcription. Additionally, we uncovered the involvement of SbWRKY41 in JA-regulated baicalin biosynthesis through its transcriptional regulation of *SbMYC2.1*, *SbJAZ3*, and *SbWRKY75* (**Figure 7**).

### JA-activated SbWRKY75 and SbWRKY41 directly enhance the expression of baicalin biosynthesis pathway genes

The construction of gene regulatory networks is a powerful approach for identifying TF-gene interactions in diverse biological contexts, and WGCNA has demonstrated superior performance in detecting functionally associated gene pairs through examination of pairwise correlations in gene expression patterns (Kuang et al., 2021). In this study, we performed a transcriptome analysis of control *S. baicalensis* roots and of roots exposed to MeJA treatment for 1, 3, or 7 h to establish a co-regulated gene network associated with baicalin biosynthesis. Through this analysis, we identified nine TF genes and their corresponding regulatory pathways responsible for baicalin biosynthesis. We confirmed the validity of the network through Y1H assays, EMSAs, and dual-LUC assays, which demonstrated that SbWRKY75, SbWRKY41, and SbRAP2.1 bound to the promoters of their respective target genes.

We showed that SbWRKY75 activated transcription from the *SbCLL-7* promoter, while SbWRKY41 activated that from two baicalin biosynthesis pathway genes. Our investigation indicated that the overexpression and RNA interference of *SbWRKY75* in transiently transformed seedlings significantly influenced the expression of baicalin biosynthetic genes and the accumulation of baicalin. Similarly, TcWRKY1 is a TF encoded by a gene whose expression is induced by MeJA and enhances the expression of *DBAT* when overexpressed in *Taxus chinensis* suspension cells. Moreover, RNAi-mediated knockdown of *TcWRKY1* transcript levels decreases *DBAT* transcript levels, thereby decreasing paclitaxel biosynthesis (Li et al., 2013). We established here that SbWRKY75 acts as a positive regulator of baicalin biosynthesis by modulating the expression of genes involved in its biosynthetic pathway. *SbWRKY75* overexpression may affect the expression of *SbCLL-7* or modulate other signaling pathways, thereby either directly or indirectly influencing the expression of baicalin biosynthesis pathway genes. However, as plants seek to maintain a balance between growth, development, and secondary metabolite biosynthesis, negative feedback regulation can inhibit gene expression when the concentration of secondary metabolites is too high. Thus, some baicalin pathway genes in *S. baicalensis* may experience a more modest decrease or change in their expression levels. Previous studies have demonstrated that WRKY TFs are closely associated with anthocyanin accumulation in *S. baicalensis* (Zhang et al., 2022). Our findings suggest that both SbWRKY75 and SbWRKY41 transcriptionally activate the expression of baicalin biosynthesis genes, thereby positively regulating baicalin accumulation.

### SbWRKY41 is essential for JA-mediated regulation of baicalin biosynthesis

In addition, we demonstrated that SbWRKY41 also regulates the expression of *SbMYC2.1* and *SbJAZ3*. MYC2 and JAZ proteins are key regulators of the JA signaling pathway, interacting with TFs that control plant secondary metabolism (Li et al., 2022). JA induces the expression of *WRKY* TF genes and regulates the activity and stability of their encoded proteins through various modifications, including phosphorylation and acetylation (Mao et al., 2007; Liao et al., 2016). Furthermore, several WRKY TFs have been implicated in the JA signaling pathway, such as WRKY40a in peppers (*Capsicum annuum*), which directly regulates the expression of *CaJAZ8* to modulate JA signaling (Raffeiner et al., 2022), and GhMYC2, which serves as a link between the WRKY–MAPK cascade and flavonoid biosynthesis in upland cotton (*Gossypium hirsutum*) (Wang et al., 2022a). Given the interaction between SbWRKY41, SbMYC2.1, and SbJAZ3, we speculate that JA-responsive SbWRKY41 also regulates baicalin biosynthesis *via* the JA signaling pathway in *S. baicalensis*.

Both the Y1H assay and EMSA revealed potential interactions between SbWRKY41 and SbWRKY75, suggesting that these two TFs may form a regulatory network to optimize the signal transduction and cooperatively regulate baicalin biosynthesis. In plants, WRKY TFs interact and cross-regulate one another, and many possess self-regulatory feedback (Turck et al., 2004). For instance, WRKY6 can inhibit its own activity by binding to the W-box in the promoter of its encoding gene and that of *WRKY42* in Arabidopsis (Robatzek and Somssich, 2002). Similar observations have been reported for CaWRKY6 and CaWRKY40 in pepper, with *CaWRKY6* showing functional and expression similarities with *CaWRKY40* (Cai et al., 2015). Based on the regulation of *SbWRKY75* by SbWRKY41, it is reasonable to speculate that SbWRKY41 and SbWRKY75 may functionally interact and form a transcriptional network to achieve optimal coordination and fine-tuning of baicalin biosynthesis.

Based on the above findings, we propose a mechanistic model outlining the possible mode of action of SbWRKY75 and SbWRKY41 in regulating the biosynthesis of baicalin in *S. baicalensis*. In the presence of bioactive JAs, SbWRKY75 and SbWRKY41 are activated, with SbWRKY41 in turn regulating the expression of *SbWRKY75*. Additionally, SbWRKY41 participates in the JA signaling pathway by modulating the expression of *SbMYC2.1* and *SbJAZ3*. By binding to the W-box *cis*-elements present in the promoters of baicalin biosynthetic genes, SbWRKY75 and SbWRKY41 activate the transcription of these genes. Our findings provide evidence for a positive regulatory role for SbWRKY75 and SbWRKY41 in baicalin biosynthesis in *S. baicalensis*, thereby offering a promising strategy for enhancing the production of baicalin through genetic engineering of specific TF genes.

## Data availability statement

The datasets presented in this study can be found in online repositories. The names of the repository/repositories and accession number(s) can be found in the article/**Supplementary Material**.

## Author contributions

SF, ZL, KC and WC conceived and designed the experiments. SF, CZ and SQ performed the experiments. SF wrote the paper. YX, ZL, KC and WC revised the manuscript. All authors contributed to the article and approved the submitted version.
