# Corrigendum: SbWRKY75- and SbWRKY41- mediated jasmonic acid signaling regulates baicalin biosynthesis

**DOI:** 10.3389/fpls.2023.1302112

**Published:** 2023-11-08

**Authors:** Shiyuan Fang, Chen Zhang, Shi Qiu, Ying Xiao, Kaixian Chen, Zongyou Lv, Wansheng Chen

**Affiliations:** ^1^ The State Administration of Traditional Chinese Medicine (SATCM) Key Laboratory for New Resources & Quality Evaluation of Chinese Medicine, Institute of Chinese Materia Medica, Shanghai University of Traditional Chinese Medicine, Shanghai, China; ^2^ Institute of Chinese Materia Madica, Shanghai University of Traditional Chinese Medicine, Shanghai, China; ^3^ Department of Pharmacy, Changzheng Hospital, Second Military Medical University, Shanghai, China

**Keywords:** JA, Baicalin, Scutellaria baicalensis Georgi, SbWRKY75, SbWRKY41

In the published article, there was an error in the **Funding** statement, page 11. The authors forgot to add an important funding body which should be ranked first.

The correct Funding statement appears below.

**Funding**

This work was funded by the National Natural Science Foundation of China (81830109), National Key Research and Development Program of China (2022YFC3501700), the Shanghai local Science and Technology Development Fund Program guided by the Central Government (YDZX20203100002948) and Young Elite Scientists Sponsorship Program by Cast (2021-QNRC1-02).

The authors apologize for this error and state that this does not change the scientific conclusions of the article in any way. The original article has been updated.

