# Supplementary figures and images for: SbWRKY75- and SbWRKY41-mediated jasmonic acid signaling regulates baicalin biosynthesis
Source: Front Plant Sci. 2023 Jun 21;14:1213662. doi: 10.3389/fpls.2023.1213662 (PMC10320291; doi:10.3389/fpls.2023.1213662)

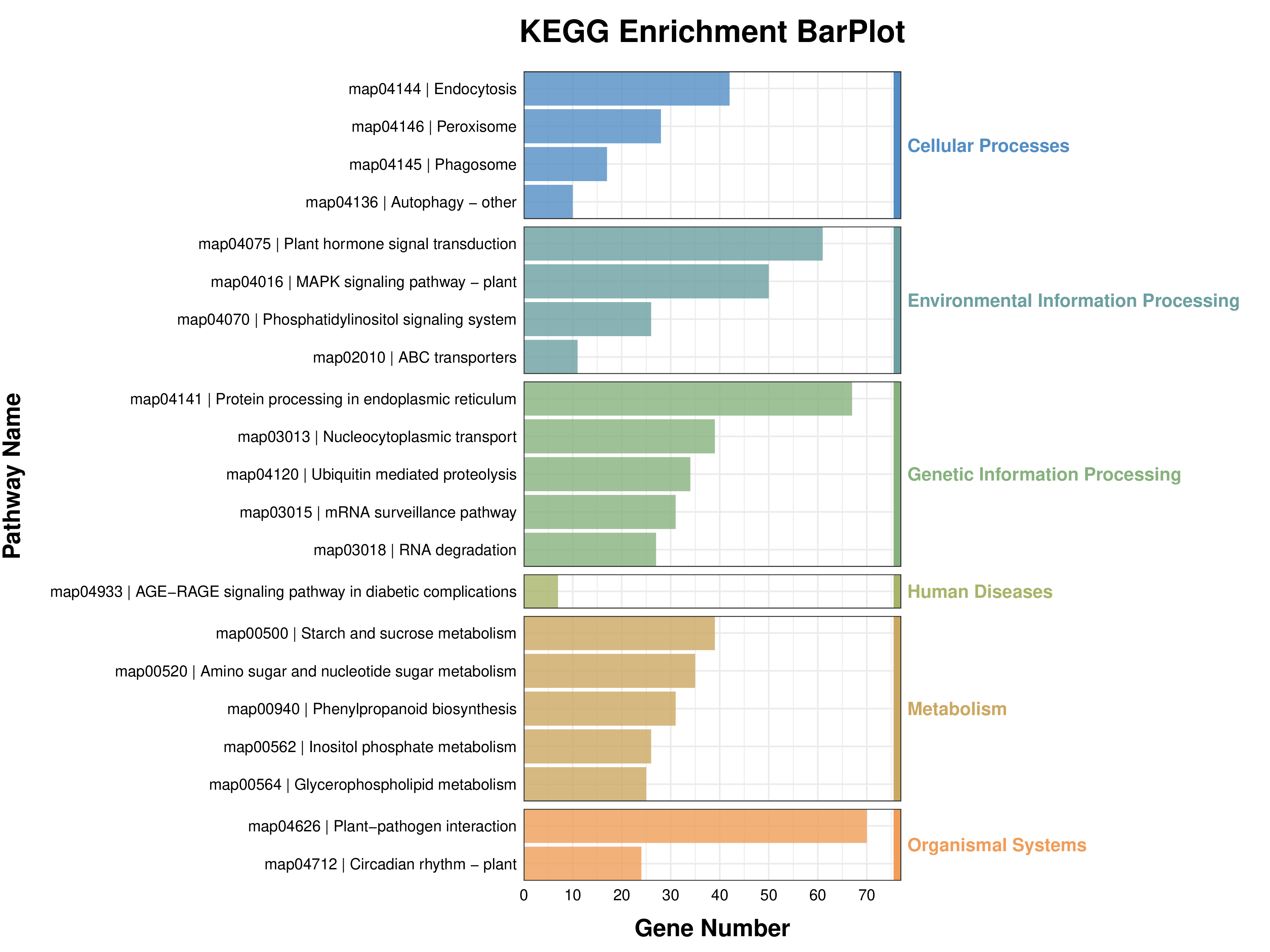

Supplement: Supplementary file 1 [file Image_1.png]

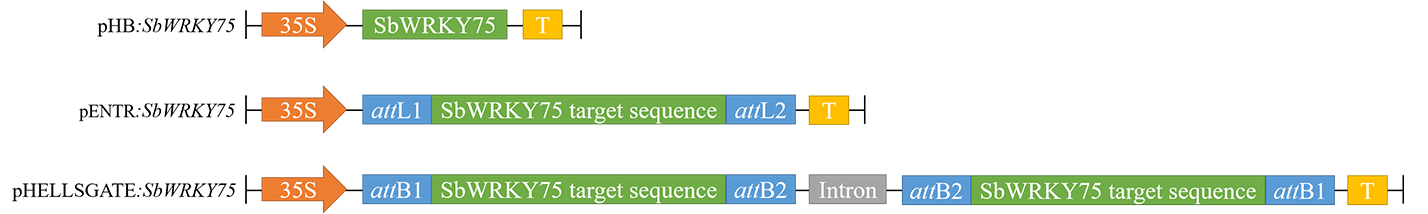

Supplement: Supplementary file 2 [file Image_2.tif]

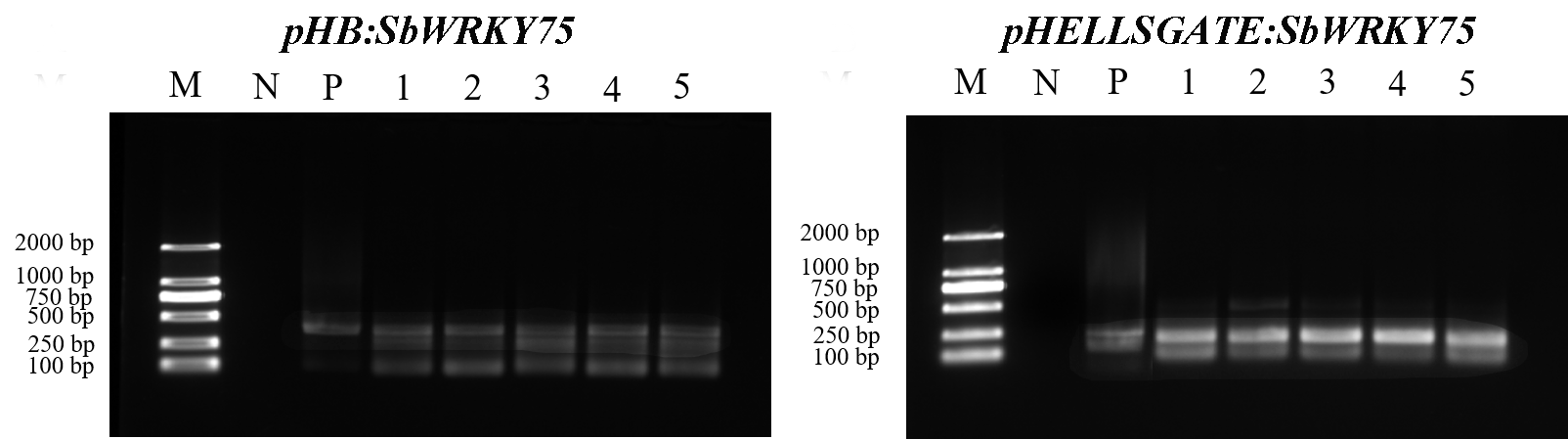

Supplement: Supplementary file 3 [file Image_3.tif]
