# Supplementary material for: SbWRKY75- and SbWRKY41-mediated jasmonic acid signaling regulates baicalin biosynthesis
Source: Front Plant Sci. 2023 Jun 21;14:1213662. doi: 10.3389/fpls.2023.1213662 (PMC10320291; doi:10.3389/fpls.2023.1213662)
Supplement: Supplementary file 4 [file Table_1.docx]

Supplement Table S1 Primers used in this study. Underlined sequences mean recombination cites for one step cloning.

| Names | Used for | Sequences |
| --- | --- | --- |
| SbCHI QPCR F | QPCR | TTCGCCATCCGTCACCAAAG |
| SbCHI QPCR R | QPCR | TCGTCTGACTCCGTCAACTCCTC |
| SbCHS-2 QPCR F | QPCR | GCCCAGAAGGCGATCAAGGAA |
| SbCHS-2 QPCR R | QPCR | ATCTCGGAGCAGACGACAAGAACC |
| SbCLL-7 QPCR F | QPCR | ATTGAGGCCACCGTTGTATC |
| SbCLL-7 QPCR R | QPCR | CGACTTTTGAAACCGTGGAT |
| SbF6H QPCR F | QPCR | TCCGCCTCCGACAAACTTCC |
| SbF6H QPCR R | QPCR | CCAGTATGGTCCGTAGGGTGAGAAC |
| SbFNSII-2 QPCR F | QPCR | TCGTCACCTATGGCGTCTCCTTC |
| SbFNSII-2 QPCR R | QPCR | ACTCGGCGGCTCTGATGCTG |
| SbGUS QPCR F | QPCR | TCAGGTTTGGCAAAAGGGGT |
| SbGUS QPCR R | QPCR | AGTTGCACTTGGTAGGAGGC |
| SbPAL-1 QPCR F | QPCR | TTGGGAAAGACTCAGATGC |
| SbPAL-1 QPCR R | QPCR | TCAAACCTAATGCCAGAATA |
| SbPAL-2 QPCR F | QPCR | TGCGTCCAACTCAGTGATCC |
| SbPAL-2 QPCR R | QPCR | GCGACCTGAGCGATAGTCAA |
| SbPAL-3 QPCR F | QPCR | TTGTCCGAATCAACACCCTCCT |
| SbPAL-3 QPCR R | QPCR | ACCAGTGCCAGCCCTTCCTTA |
| SbUGT QPCR F | QPCR | ATCGGCAGGCATCCTCGTTAA |
| SbUGT QPCR R | QPCR | TGTATCAAGCCATCTCAGACACTCATG |
| SbWRKY75 QPCR F | QPCR | CTATCCAGCAGCCATGTTCC |
| SbWRKY75 QPCR R | QPCR | TTCGCCTCAATTCCGACCAC |
| SbACT7 QPCR F | QPCR | CAGGAAGCAGAAACAGCAAAGA |
| SbACT7 QPCR R | QPCR | CAATGATGGCTGGAACAACACT |
| SbWRKY75 pet F | Inducible protein | GGATCCGAATTCGAGCTCATGGATAACTATCCAGCAGC |
| SbWRKY75 pet R | Inducible protein | GAGTGCGGCCGCAAGCTTAAGAGAAGTATAAATTTGCA |
| SbWRKY41 pet F | Inducible protein | GGATCCGAATTCGAGCTCATGGAGAGTGCTTCTTCTTC |
| SbWRKY41 pet R | Inducible protein | GAGTGCGGCCGCAAGCTTTGTGAAAAATCCAGACATGT |
| SbRAP2.1 pet F | Inducible protein | GGATCCGAATTCGAGCTCATGGAAGGAGTGTACTGTAC |
| SbRAP2.1 pet R | Inducible protein | GAGTGCGGCCGCAAGCTTGTTCTCCTCGGAGCTTTCAG |
| SbWRKY75 Overexpression F | Overexpression | CGGGCCATGAATTCCTGCAGATGGATAACTATCCAGCAGC |
| SbWRKY75 Overexpression R | Overexpression | GCTCTAGAACTAGTGGATCCAAGAGAAGTATAAATTTGCA |
| SbWRKY75 RNAi F | RNAi | CCAACTTCTTCTTCTGTACAG |
| SbWRKY75 RNAi R | RNAi | CCTAGGTTTTCTCGACTTCTTC |
| SbWRKY75 pB42AD F | Y1H | ATTATGCCTCTCCCGAATTCATGGATAACTATCCAGCAGC |
| SbWRKY75 pB42AD R | Y1H | GAAGTCCAAAGCTTCTCGAGAAGAGAAGTATAAATTTGCA |
| SbWRKY41 pB42AD F | Y1H | ATTATGCCTCTCCCGAATTCATGGAGAGTGCTTCTTCTTC |
| SbWRKY41 pB42AD R | Y1H | GAAGTCCAAAGCTTCTCGAGTGTGAAAAATCCAGACATGT |
| SbRAP2.1 pB42AD F |  | ATTATGCCTCTCCCGAATTCATGGAAGGAGTGTACTGTAC |
| SbRAP2.1 pB42AD R |  | GAAGTCCAAAGCTTCTCGAGGTTCTCCTCGGAGCTTTCAG |
| proSbCHI LacZ F | Y1H | TTGATATTGGATCGGAATTCCTTATTTTTAGAGTTTATAA |
| proSbCHI LacZ R | Y1H | TACAGAGCACATGCCTCGAGGTTGATTATGGATTGATAGG |
| proSbCHS-2 (1) LacZ F | Y1H | TTGATATTGGATCGGAATTCTCTCTCCAGCAAGCATCAAA |
| proSbCHS-2 (1) LacZ R | Y1H | TACAGAGCACATGCCTCGAGTGTCGCCGGAGAAGAGGTAA |
| proSbCHS-2 (2) LacZ F | Y1H | TACAGAGCACATGCCTCGAGCTTGATCGACAAGAGGAGGA |
| proSbCHS-2 (2) LacZ R | Y1H | TACAGAGCACATGCCTCGAGTGTCGCCGGAGAAGAGGTAA |
| proSbCHS-2 (3) LacZ F | Y1H | TTGATATTGGATCGGAATTCCACGATACACCTTTCATGAG |
| proSbCHS-2 (3) LacZ R | Y1H | TACAGAGCACATGCCTCGAGTGTCGCCGGAGAAGGAGGTA |
| proSbCLL-7 LacZ F | Y1H | TTGATATTGGATCGGAATTCTTTTAAATGTCTGGGATGAG |
| proSbCLL-7 LacZ R | Y1H | TACAGAGCACATGCCTCGAGCTTCTTCCTCCTTTTTCTGG |
| proSbF6H LacZ F | Y1H | TTGATATTGGATCGGAATTCCTCATATATAATATTTTAAA |
| proSbF6H LacZ R | Y1H | TACAGAGCACATGCCTCGAGGGATGTGTGAGTGAGTGAGT |
| proSbFNSII-2 LacZ F | Y1H | TTGATATTGGATCGGAATTCTCTACTTTTCAGATAAGGTG |
| proSbFNSII-2 LacZ R | Y1H | TACAGAGCACATGCCTCGAGCGGGATTACAGTTGCTATTT |
| proSbGUS LacZ F | Y1H | TTGATATTGGATCGGAATTCTGATAATAATTTAGCGACAA |
| proSbGUS LacZ R | Y1H | TACAGAGCACATGCCTCGAGCTTCAACGAGAAAAGATCAA |
| proSbPAL-1 LacZ F | Y1H | TTGATATTGGATCGGAATTCCGCATCGGTTGAAGAAGAAG |
| proSbPAL-1 LacZ R | Y1H | TACAGAGCACATGCCTCGAGTCTTCTAAAAATAGTGATTC |
| proSbPAL-2 LacZ F | Y1H | TTGATATTGGATCGGAATTCATGAAGCGAAACATGGCACT |
| proSbPAL-2 LacZ R | Y1H | TACAGAGCACATGCCTCGAGGATCCGATGAGAAGCAGGAT |
| proSbPAL-3 LacZ F | Y1H | TTGATATTGGATCGGAATTCGCCAAATCTTTCCAGCTTAA |
| proSbPAL-3 LacZ R | Y1H | TACAGAGCACATGCCTCGAGTGCAAGAAAGAGTGAAGAAC |
| proSbUGT LacZ F | Y1H | TTGATATTGGATCGGAATTCGATCTCAATCTAAAACGAAA |
| proSbUGT LacZ R | Y1H | TACAGAGCACATGCCTCGAGTTTTTGATAGATAGATAGGT |
| proSbWRKY75 LacZ F | Y1H | TTGATATTGGATCGGAATTCGGGATGTTTTGGAGTAACAC |
| proSbWRKY75 LacZ R | Y1H | TACAGAGCACATGCCTCGAGGAGAGAGAAAGGAAGAATGA |
| proSbMYC2.1 LacZ F | Y1H | TTGATATTGGATCGGAATTCCAATTTCATTGTCCACGACT |
| proSbMYC2.1 LacZ R | Y1H | TACAGAGCACATGCCTCGAGGGGCGAGGGTGAGCAGAGAG |
| proSbJAZ3 LacZ F | Y1H | TTGATATTGGATCGGAATTCCTGTGGCCACAATCAGGGTC |
| proSbJAZ3 LacZ R | Y1H | TACAGAGCACATGCCTCGAGCTCTCTCTCCGGCGACAAAA |
| SbWRKY41 pHB F | Dual-LUC | CGGGCCATGAATTCCTGCAGATGGAGAGTGCTTCTTCTTC |
| SbWRKY41 pHB R | Dual-LUC | GCTCTAGAACTAGTGGATCCTGTGAAAAATCCAGACATGT |
| proSbCLL-7 pGreenII 0800 F | Dual-LUC | TCGACGGTATCGATAAGCTTTTTTAAATGTCTGGGATGAG |
| proSbCLL-7 pGreenII 0800 R | Dual-LUC | GTGGATCCCCCGGGCTGCAGCTTCTTCCTCCTTTTTCTGG |
| proSbF6H pGreenII 0800 F | Dual-LUC | TCGACGGTATCGATAAGCTTCTCATATATAATATTTTAAA |
| proSbF6H pGreenII 0800 R | Dual-LUC | GTGGATCCCCCGGGCTGCAGGGATGTGTGAGTGAGTGAGT |
| proSbUGT pGreenII 0800 F | Dual-LUC | TCGACGGTATCGATAAGCTTGATCTCAATCTAAAACGAAA |
| proSbUGT pGreenII 0800 R | Dual-LUC | GTGGATCCCCCGGGCTGCAGTTTTTGATAGATAGATAGGT |

Supplement Table S2 Number of genes in modules derivied from WGCNA.

| Modules | Numbers |
| --- | --- |
| module_black | 749 |
| module_blue | 4052 |
| module_brown | 3364 |
| module_cyan | 227 |
| module_green | 877 |
| module_greenyellow | 420 |
| module_grey | 234 |
| module_magenta | 528 |
| module_pink | 583 |
| module_purple | 492 |
| module_red | 778 |
| module_salmon | 232 |
| module_tan | 396 |
| module_turquoise | 9559 |
| module_yellow | 1099 |
